# Liberality of a Macon Drug House

**Published:** 1884-12-20

**Authors:** 


### LIBERALITY OF A MACON DRUG HOUSE.

Messrs. Goodwyn & Small, pharmaceutists and druggists, of Macon, Georgia, have donated to the Ivy street Hospital of this city a lot of their new and valuable preparation of the Compound Syrup of the Hypophosphites. The physicians in charge of the Hospital are much pleased with the preparation, and warmly appreciate the kindness of the generous donors.

